# When History Returns: Hybrid Management of Syphilitic Aortitis Causing Severe Aortic Arch Branch Stenosis and Cerebral Hypoperfusion

**DOI:** 10.1016/j.ejvsvf.2026.10.001

**Published:** 2026-10-16

**Authors:** Julia Al Mousli, Anders Håkanson, Jonas Malmstedt, David Lindström

**Affiliations:** a Department of Surgery, Section of Vascular Surgery, Södersjukhuset, Stockholm, Sweden; b Doctors of the World, Stockholm, Sweden; c Department of Clinical Science and Education, Södersjukhuset, Karolinska Institutet, Stockholm, Sweden

**Keywords:** Aortitis, Cardiovascular syphilis, Carotid stenosis, Carotid to subclavian bypass, Cerebral ischaemia

## Abstract

**Introduction:**

Cardiovascular manifestations of tertiary syphilis are rare in the antibiotic era, but important to consider in atypical vascular findings. These manifestations typically appear several years after the primary infection and may lead to structural damage in the cardiovascular system, requiring surgical intervention despite appropriate antibiotic therapy.

**Report:**

This case describes a rare manifestation of cardiovascular syphilis in a 46 year old woman presenting with dizziness, pre-syncope, and syncope consistent with global cerebral ischaemia. Extensive evaluation revealed syphilitic aortitis with severe stenosis and or occlusion of all the major aortic arch branches. The patient was managed with antibiotic therapy in combination with surgical revascularisation. Both endovascular and open surgical approaches were used to restore blood flow to the left common carotid, left vertebral, and left subclavian arteries. This intervention improved cerebral perfusion and resolved the patient’s symptoms. At two years, the carotid stent was patent, while the carotid subclavian bypass was occluded. The patient remained free from cerebral ischaemic symptoms but reported bilateral arm claudication.

**Conclusion:**

Tertiary syphilis should be considered as a differential diagnosis when atypical arterial disease is encountered. Hybrid revascularisation may provide an effective, less invasive treatment strategy.

## HIGHLIGHTS

• Aortitis from tertiary syphilis caused critical stenoses in all aortic arch branches.
• Cerebral hypoperfusion and arm claudication resulted from syphilitic arteriopathy.
• Hybrid open and endovascular revascularisation was performed successfully.
• This rare case underscores the need to consider infectious causes in vascular disease.

## INTRODUCTION

Syphilis, caused by the spirochete *Treponema pallidum* (*T. pallidum*), is a chronic sexually transmitted infection. After decades of declining incidence, global rates have increased, with the World Health Organisation estimating eight million new infections among adults aged 15 – 49 years in 2022.^1^, ^2^

The infection can have three stages: primary, secondary, and tertiary. In some untreated patients the infection resolves after one stage, and in others it enters a latent stage that can later become active if left untreated. Tertiary syphilis develops in approximately one third of untreated patients, usually after a latent period of at least five years. Syphilitic aortitis, usually of the thoracic aorta, is the most common cardiovascular manifestation of tertiary syphilis, with an incidence of ∼10% among untreated patients, manifesting 10 – 40 years after the initial infection.^2^, ^3^ The pathogenesis involves *T. pallidum* invading the aortic wall and triggering a destructive inflammatory response, leading to scarring and weakening of the aortic wall.^4^

Non-treponemal serological tests, such as the Rapid Plasma Reagin (RPR) and Venereal Disease Research Laboratory (VDRL) tests, have been used for screening, but they have been replaced by antibody tests. The non-treponemal tests are mainly used for follow up after treatment. The treponemal tests, particularly the *T*. *pallidum* haemagglutination assay (TPHA) or the *T*. *pallidum* particle agglutination assay (TPPA), are used for confirmation; these tests also remain positive after successful treatment.^3^

Penicillin remains the standard therapy for all stages of infection. For cardiovascular forms of tertiary syphilis, intramuscular benzathine penicillin G administered on three occasions one week apart is recommended if there are no signs of neuronal involvement in the lumbar puncture.^3^

This case describes a rare complication of syphilitic aortitis, characterised by severe stenosis and or occlusion of all major aortic arch branches. Written informed consent for publication of this case report and accompanying images was obtained from the patient.

## REPORT

A 46 year old woman from another European country, living part time in Sweden, with no relevant past medical history other than active smoking, presented in 2021 with progressive fatigue, dizziness, pre-syncope, and syncope. She reported cold hands bilaterally and decreased work capacity in the arms. She had no fever or skin rash. Physical examination revealed a good general condition. She had a regular heart rhythm without heart murmurs, but a bruit was noted over the left carotid artery. Upper extremity pulses were impalpable. Arm systolic blood pressure was approximately 60 mmHg, measured by Doppler in the radial arteries bilaterally, while ankle pressure was 160 mmHg bilaterally. Sensory and motor function in both upper and lower extremities was preserved.

Blood tests, including C reactive protein, erythrocyte sedimentation rate, liver and kidney function tests, were within normal ranges. Urine sediment and autoimmune screening were negative. Electrocardiogram and echocardiography showed no abnormalities.

Computed tomography angiography (CTA) revealed a short occlusion of the brachiocephalic trunk, a high grade stenosis at the origin of the left common carotid artery, and occlusion of the left subclavian artery proximal to the origin of the vertebral artery (Fig. 1).

**Figure 1:**
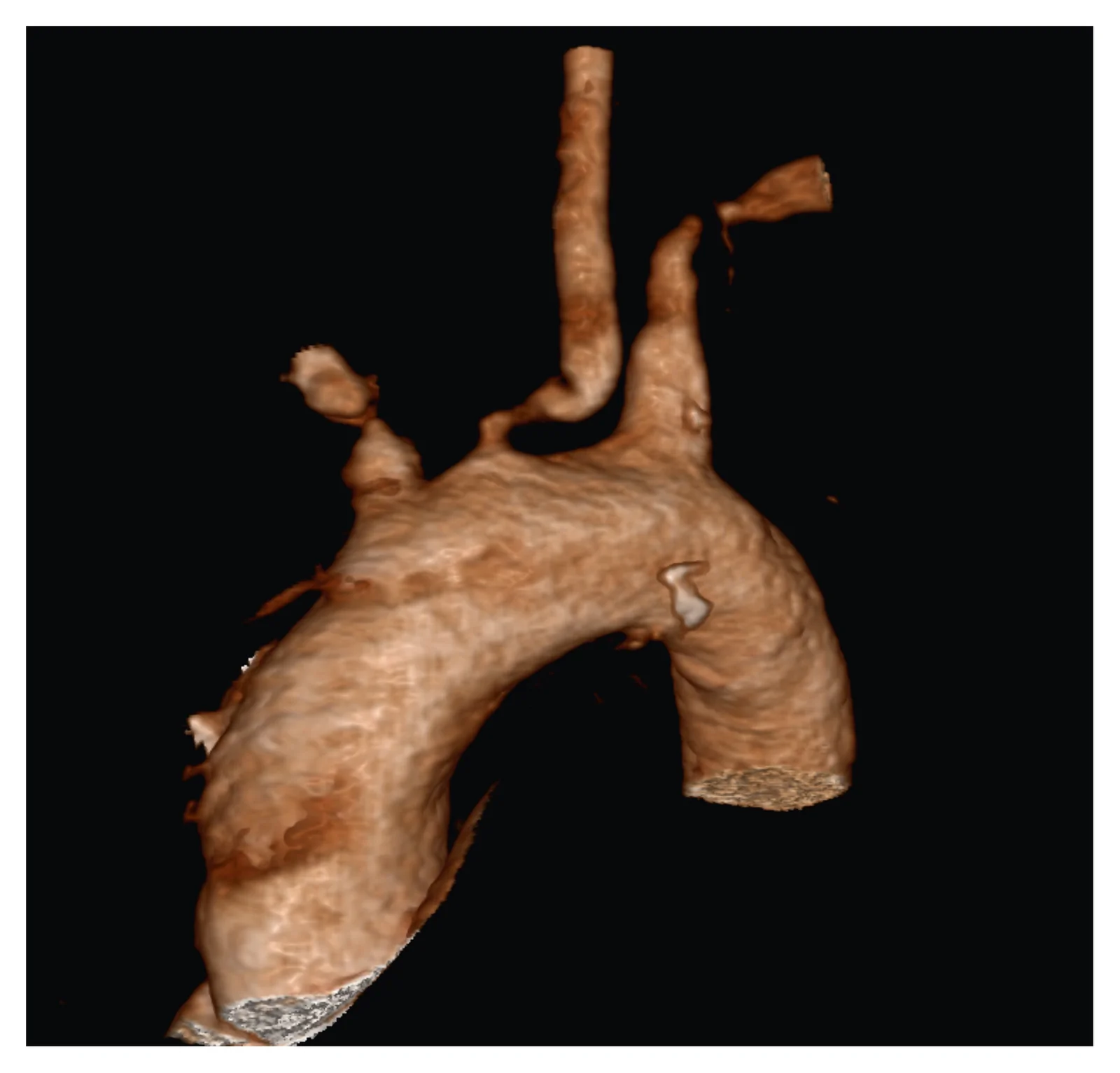
Three dimensional computed tomography angiography reconstruction demonstrating occlusive and stenotic lesions involving all three major branches of the aortic arch.

The patient was strongly advised to stop smoking. Further rheumatological evaluation was initiated to investigate the possibility of large vessel vasculitis, such as Takayasu arteritis. Ultrasound of the temporal arteries, common carotid arteries, the subclavian and axillary arteries showed no vessel wall changes consistent with large vessel vasculitis. Blood samples were also taken for syphilis, hepatitis B, and HIV.

All syphilis serological tests returned positive, consistent with tertiary syphilis, and the patient was diagnosed with syphilitic aortitis. Given the cardiovascular involvement, a lumbar puncture was performed, which ruled out central nervous system involvement. Treatment consisted of intramuscular benzathine penicillin G (2.4 million units once weekly for three weeks). Follow up serology was planned at three, six, and 12 months. Tests at three and six months were obtained, but the patient (a non-Swedish citizen) then lived abroad for almost three years and missed the planned assessments. This caused a prolonged interval before revascularisation was considered.

In December 2023, follow up syphilis serology showed essentially unchanged titres compared with 2021, which were interpreted as being consistent with a residual serological response rather than treatment failure. Nevertheless, after deliberation between the genitourinary medicine and the infectious diseases specialists it was decided, as a precautionary measure, to prescribe an additional four week course of high dose doxycycline. This decision reflected the incomplete serological follow up, as the patient had spent prolonged periods abroad, making verification of treatment response difficult. Oral doxycycline was chosen because repeat benzathine penicillin administration was not feasible for logistical reasons.

Although the infectious component in the aorta was considered treated, the patient increasingly experienced dizziness and pre-syncope. Repeat CTA confirmed persistent occlusion or high grade stenosis of all major aortic arch branches. Following multidisciplinary discussion between vascular surgery and infectious diseases, revascularisation was considered indicated because of persistent symptoms of cerebral hypoperfusion despite antibiotic treatment.

Left cerebral hemisphere and left arm revascularisation were perfomed in March 2024. The primary indication was symptomatic cerebral hypoperfusion rather than upper extremity ischaemia.

The major concern was how to avoid intra-operative cerebral hypoperfusion. Firstly, open exposure of the left common carotid and left subclavian arteries was obtained, followed by retrograde stenting of the proximal left common carotid artery using a BeGraft (Bentley InnoMed, Hechingen, Germany) stent graft. Test clamping of the left carotid showed a significant drop in near infrared spectroscopy (NIRS, Foresight (CAS Medical Systems Inc., Branford, CT, USA)). To preserve cerebral perfusion during left carotid–subclavian bypass, endovascular recanalisation of the chronically occluded brachiocephalic trunk was attempted but unsuccessful. Open reconstruction would have entailed a higher operative risk and was therefore not performed. A decision was then taken to use an endovascular shunt during carotid anastomosis. A 6 mm Dacron graft was sutured to the left subclavian artery, then a short 6 F dialysis sheath was placed in the left carotid bulb cranially and connected with a tube to the 7 F sheath going down in the left common carotid. Test clamping of the carotid artery between the sheaths then showed no significant NIRS drop. The graft was sutured to the left common carotid with a 15 minute clamp time (Fig. 2). Intra-operative angiography demonstrated patent reconstructions and collateral circulation to the right hemisphere, indicating improved cerebral perfusion bilaterally.

**Figure 2:**
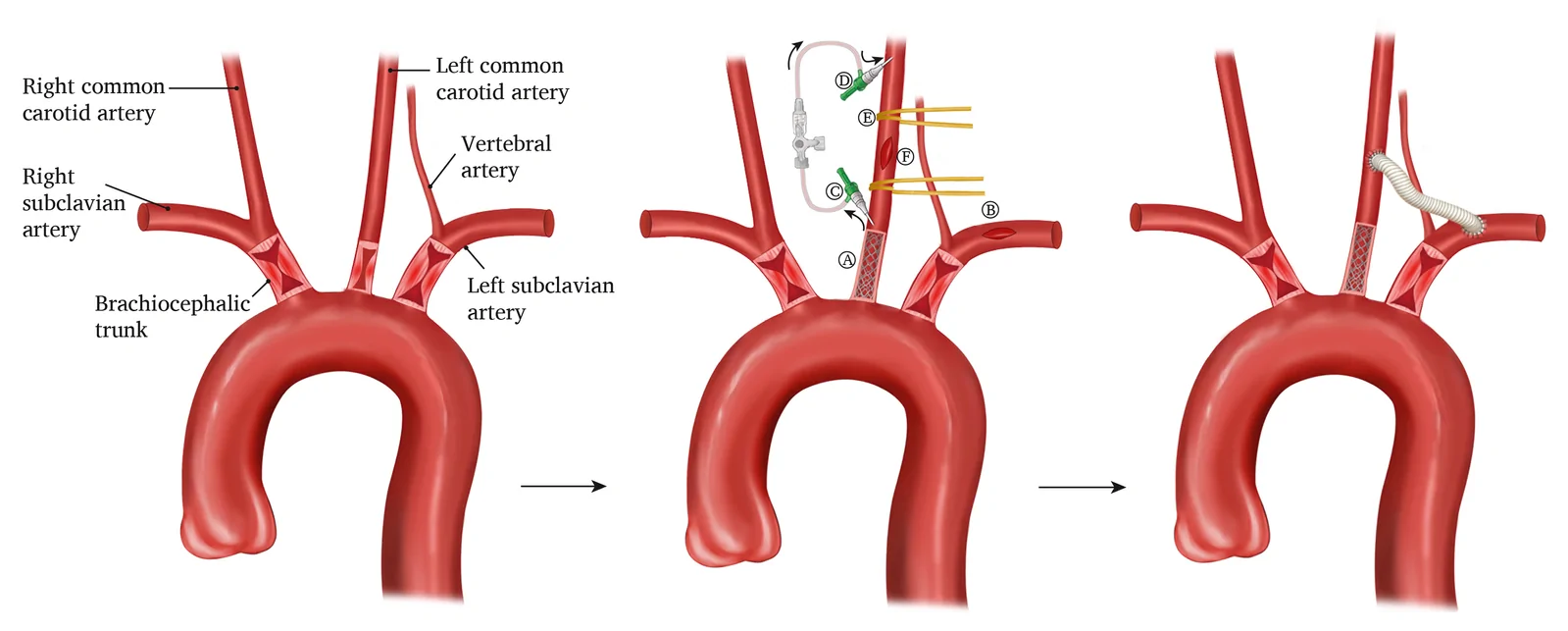
Schematic illustration of the vascular anatomy and operative steps. Left: Pre-operative anatomy. Middle: Intra-operative configuration showing A) stent in the left common carotid artery, B) intended site of anastomosis to the left subclavian artery, C) 7F sheath connected via tubing and a three way stopcock to D) 6F dialysis sheath, E) vessel loops, and F) intended site of anastomosis to the left common carotid artery. Right: final reconstruction following left common carotid artery stenting and left carotid–subclavian bypass.

Post-operatively, close blood pressure monitoring was mandated to avoid both hypo- and hyperperfusion. Blood pressure trended toward the higher range, and antihypertensive medication was initiated, as well as aspirin. She was discharged home on post-operative day five without any major complications.

At the three and nine month follow ups, the patient was neurologically intact and reported no further dizziness, pre-syncope, or syncope. Duplex ultrasound demonstrated patent reconstructions. The patient lived abroad for a period and was re-evaluated two years after surgery. She remained free from cerebral ischaemic symptoms but reported bilateral arm claudication and non-specific shoulder pain at rest. Radial systolic pressure measured by Doppler was 80 mmHg bilaterally, and ankle systolic pressure was approximately 200 mmHg. CTA demonstrated a patent left common carotid stent but an occluded carotid–subclavian bypass (Fig. 3). Revision of the occluded carotid–subclavian bypass was not pursued because cerebral symptoms had resolved and revision would have been expected to improve only left upper extremity perfusion, whereas the patient's arm symptoms were mild and bilateral.

**Figure 3:**
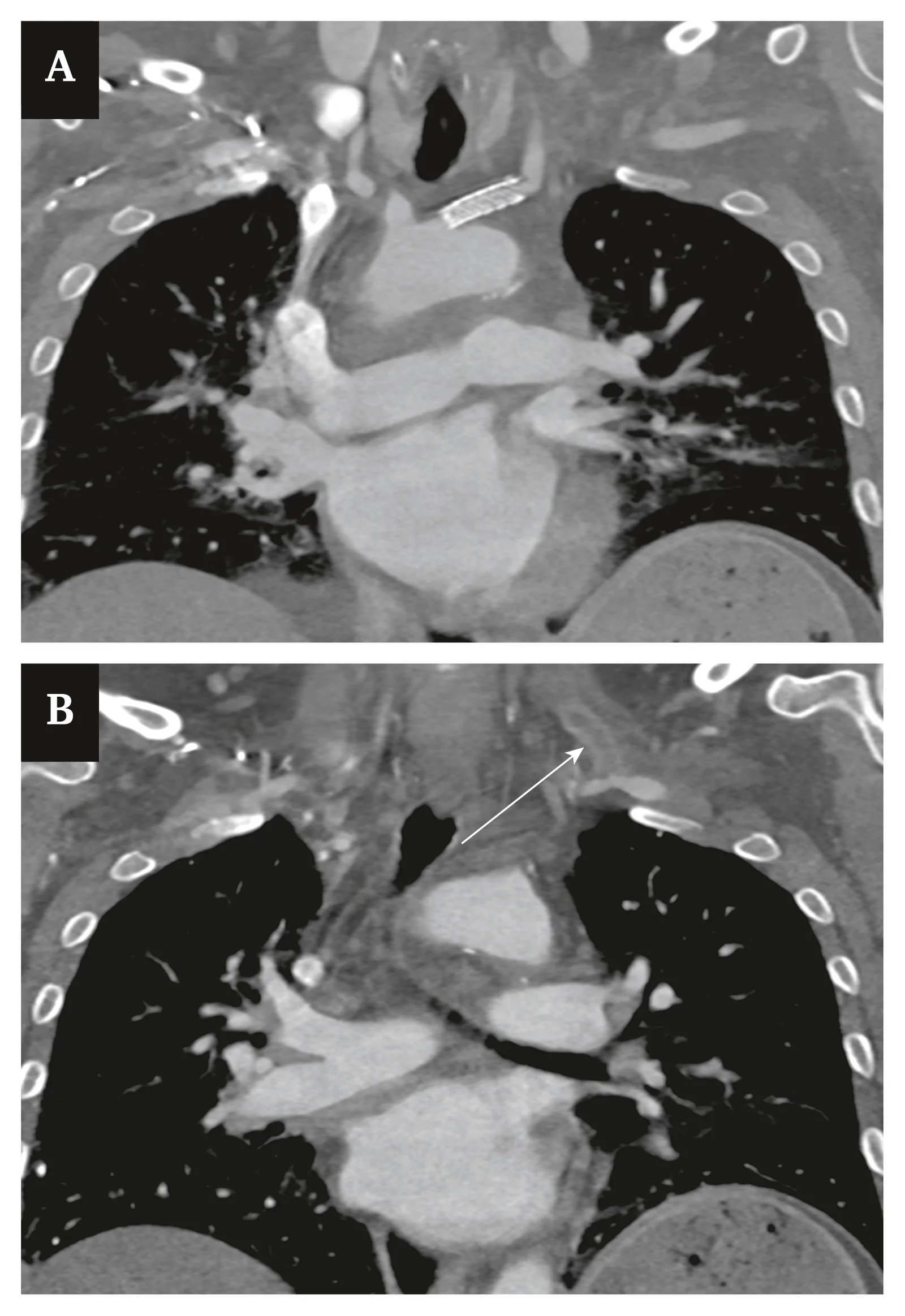
Post-operative computed tomography angiography with coronal reconstructions. A) Unchanged occlusion of the brachiocephalic trunk, and a patent left common carotid artery stent. B) Occlusion of the left carotid–subclavian bypass (white arrow).

The patient reported good compliance with her medications and had stopped smoking. Aspirin monotherapy was chosen post-operatively as it reflects institutional practice for carotid–subclavian bypasses. It is unlikely that a short course of dual antiplatelets (which would have been a plausible alternative) would have prevented the late bypass occlusion. The cause of bypass occlusion remains unclear, although a potential low flow state in the graft may have contributed to thrombosis and occlusion over time.

## DISCUSSION

Syphilitic aortitis most commonly involves the ascending and thoracic aorta and typically leads to aneurysmal degeneration.^5^, ^6^ Although occlusive vascular manifestations of tertiary syphilis have previously been reported, including coronary ostial stenosis and cerebrovascular disease, involvement of all three major aortic arch branches resulting in global cerebral hypoperfusion appears to be very rare.^7^, ^8^

The clinical and radiological findings initially mimicked large vessel vasculitis, particularly Takayasu arteritis. However, the absence of systemic inflammation, negative autoimmune screening, and lack of typical signs on duplex ultrasound argued against large vessel vasculitis.^3^, ^5^ These findings were also inconsistent with atherosclerosis, as both the proximal and distal aorta appeared normal. Positive treponemal and non-treponemal serology was key to the diagnosis, highlighting the importance of considering tertiary syphilis in atypical vascular findings and performing serological testing when the diagnosis is suspected.^4^, ^9^

Endarteritis obliterans of the vasa vasorum plays a central role in the pathophysiology of syphilitic aortitis, leading to ischaemic injury, fibrosis, and scarring of the aortic wall. The same pathological process can also cause progressive luminal narrowing, as seen in meningovascular neurosyphilis. In this condition, obliterative endarteritis of intracranial arteries leads to stenosis and ischaemic stroke. The mechanism involves narrowing of the vascular lumen due to intimal thickening from chronic inflammation and infiltration by plasma cells and lymphocytes, which is consistent with the occlusive vessel changes observed in the present case.^2^, ^10^

Despite appropriate antibiotic therapy, surgical intervention was required due to cerebral ischaemic symptoms. A purely endovascular approach was considered unsuitable due to chronic occlusion of the brachiocephalic trunk and because the subclavian lesion was very close to the vertebral artery origin, with a risk of inadvertent vertebral artery coverage. Open arch reconstruction would have been associated with significantly higher surgical risk. Therefore, a hybrid approach to restore perfusion to the left cerebral hemisphere and left arm, while minimising operative risk was chosen. The intra-operative use of NIRS and an endovascular shunt was crucial to protecting cerebral perfusion during carotid clamping.

### Conclusion

Hybrid revascularisation successfully resolved cerebral ischaemic symptoms despite later occlusion of the carotid–subclavian bypass and persistent bilateral arm claudication, illustrating both the potential benefits and limitations of hybrid management in advanced cardiovascular syphilis.

## USE OF GENERATIVE AI TOOLS

During the preparation of this work the authors used Grammarly in order to correct grammar and to check spelling. The authors take full responsibility for the content of the publication.
